# Retraction Note: Corona virus anxiety and Chinese students’ cognitive, affective, and behavioral engagement, and academic resilience: correlations and perceptions

**DOI:** 10.1186/s40359-025-02813-x

**Published:** 2025-05-06

**Authors:** Xiaoling Yang, Yanmeng Geng

**Affiliations:** 1 Prospect College, Jinzhong College of Information, 030800 Jinzhong, China; 2 School of Mudanjiang Medical University, 157011 Mudanjiang, China

**Retraction Note: BMC Psychology (2024) 12:107**

10.1186/s40359-024-01548-5

The editor and the publisher have retracted this article. The article was submitted to be part of a guest-edited issue. An investigation by the publisher found a number of articles, including this one, with a number of concerns, including but not limited to compromised editorial handling and peer review process, inappropriate or irrelevant references or not being in scope of the journal or guest-edited issue. Based on the investigation’s findings the editor therefore no longer has confidence in the results and conclusions of this article.

The authors have not responded to correspondence regarding this retraction.

